# Bellevue Medical College

**Published:** 1881-04

**Authors:** 


BELLEVUE MEDICAL COLLEGE.—Far better would it have been for Bellevue Medical College to have gone on in the even tenor of its way than to have acted under a spasmodic impulse of reform in medical education, and in a short while reverse its action, and resume its old status. It must be humiliating to all of the high toned alumni of that heretofore prominent school, to contemplate the recent action of their *Alma Mater!* One thing is made clearly manifest in this retrograde action, viz: That money, or the lack of it, was its controlling motive. We feel deeply pained to record this back down of the faculty, and especially at this juncture, when real, earnest efforts are being made for the elevation of the profession by prolonging the term of study in the colleges, and otherwise by several schools, some of which are pecuniarily less strong than Bellevue Medical College is, or was. This failure to stand up and battle for the right, prominent as this college is, should not be allowed to weaken the faith, nor lessen the efforts of those schools which have determined that their diplomas shall have more value than a mere licence to practice medicine, and collect fees attached to them. There are some schools which can grant diplomas, and confer degrees that will doubtless rejoice to have Bellevue Medical College to lean upon and strengthen them before the public, in their short and easy terms, but unless we greatly mistake the signs of the times, short sessions, and "Three years study, including two courses of lectures" have reached already their largest pecuniary returns.

We feel assured that those institutions which will buckle on their armour afresh and press onward in the good fight for elevating the profession, will ere long meet a grand reward, and the plaudit of well done, by the profession and intelligent laity of our whole country.

